# Quantum mechanical and machine learning prediction of rotational energy barriers in halogenated aromatic alcohols

**DOI:** 10.1007/s00894-025-06321-y

**Published:** 2025-02-24

**Authors:** Steven T. Cerabona, Gordon G. Brown, Leah B. Casabianca

**Affiliations:** 1 Department of Chemistry, Clemson University, Clemson, SC 29634 USA; 2 South Carolina Governor’s School for Science and Mathematics, Hartsville, SC 29550 USA

**Keywords:** Rotational energy barrier, Substituent, Halogen, Aromatic alcohols, Machine learning

## Abstract

**Context:**

Rotation about a chemical bond is important in many chemical processes and can be influenced by neighboring substituents on a molecule. Rotational energy barriers can be predicted by density functional theory (DFT) calculations. Here, we specifically explore how substituents influence the barrier to rotation about the C-O bond in symmetrically halogenated aromatic alcohols. A machine learning model was trained on the DFT-calculated rotational energies and was found to do a good job predicting rotational energy barriers from the electronegativity, atomic radius, and Hammett constant for each substituent. The machine learning model was found to perform better when it was trained separately on pyrenols, anthranols, or phenols than when it was trained on all classes of compounds together. Even though the models were trained on compounds containing only one kind of substituent, they were found to perform similarly well on compounds containing mixed substituents. Machine learning was able to predict the rotational energy barrier heights better than correlations among parameters that would be expected to be relevant based on chemical intuition.

**Methods:**

DFT calculations were done with Gaussian 16 software at the B3LYP/6–311 + G(d.p) level of theory. Machine learning was done using the classification and regression training (caret) package in R version 4.4.0.

**Supplementary Information:**

The online version contains supplementary material available at 10.1007/s00894-025-06321-y.

## Introduction

Rotation about a chemical bond is crucial to many important chemical processes, including enzymatic reactions [1], conversion between active and inactive forms of active pharmaceutical ingredients (APIs) [2], and the function of molecular switches [3]. Many factors are expected to influence the energy required to rotate a bond about a dihedral angle, including the bond order, steric effects, and stabilizing interactions with nearby atoms. Being able to predict the rotational energy barrier about a certain chemical bond would allow chemists to tune this energy barrier to facilitate or suppress selected chemical processes of interest.

Previous studies have examined the effect of nearby substituents on rotational energy barriers. Substituents can influence rotational energy barriers through both inductive [4] and steric [5] effects. Computational studies have been used to understand how substituents present on organic linkers influence the rotational energy barrier in metal–organic frameworks (MOFs) [6]. Tanaka and Okuyama [7] experimentally found a relationship between the Hammett constant of the substituent and the change in rotational energy barrier upon excitation for a series of meta-substituted toluenes. Substituents can affect other properties of molecules as well. For example, Bauzá et al. [8] have used density functional theory (DFT) calculations to understand the effect of substituents on halogen binding strength in aromatic compounds. Machine learning has been used to predict the effect of substituents on activation energies [9, 10], bond dissociation energies [11, 12], products of Diels–Alder reactions [13], redox activities [14], favored reaction pathways [15], electronic properties [16], and nonlinear optical properties [17].

Quantum mechanical tunneling can be observed in microwave spectroscopy as a splitting of rotational energy transition lines [18–25]. In particular, molecules have often been observed to tunnel through a symmetric potential energy barrier, where the energy minima on both sides of the barrier are degenerate. One example of such a rotational energy barrier is the rotation of the dihedral angle about the C-O bond in an aromatic alcohol such as phenol [26, 27]. The two energy minima occur at dihedral angles of 0° and 180°, where the O–H bond lies in the plane of the aromatic molecule. Symmetrically substituted phenols have been observed to exhibit quantum tunneling in microwave spectroscopy experiments (Cerabona and Brown, unpublished results).

The aim of this paper is to examine a series of halogenated aromatic alcohols that might be candidates for quantum tunneling between the two local minima of the -OH position. The potential energy for rotation about the C-O bond was calculated using DFT methods. We have not considered the width of the potential energy barrier in these studies, only the barrier height. The results of the DFT calculations were then used to train a machine learning model, which could be used to predict the barrier height to rotation based on the electronegativity, atomic radius, and Hammett constant of each substituent. The machine learning model was found to do a better job at predicting the barrier height to rotation than correlations between other, perhaps more intuitive, parameters such as the C-O bond order or the π-electron density at the -OH carbon. Results of this study could be useful for predicting rotational energy barriers of other substituted aromatic compounds, as well as understanding the performance of machine learning toward prediction of chemical properties of molecules.

## Methods

**Fig. 1:**
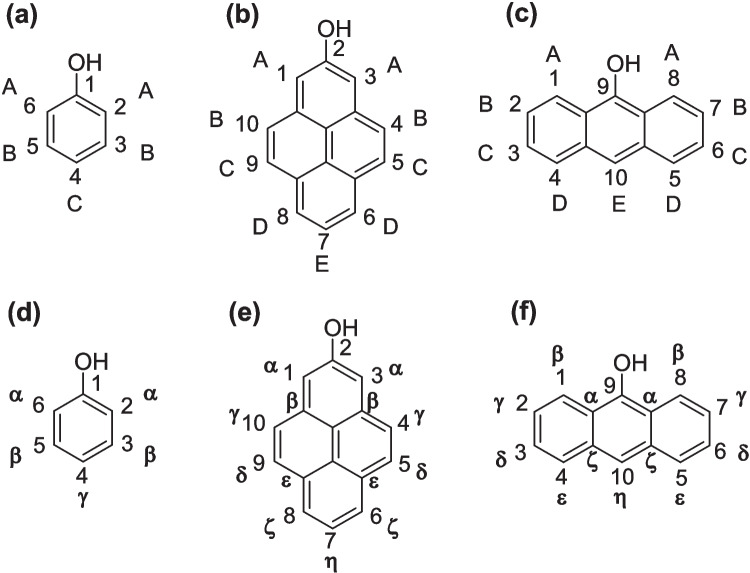
Structure of phenol (**a**, **d)**, pyren-2-ol (**b**, **e**), and anthran-9-ol (**c**, **f**) with IUPAC numbering of substituents. Because only symmetric compounds were considered in this study, we have also introduced two different labeling schemes for symmetrical substituents. The first labeling scheme (**a**–**c**) labels each available substituent position sequentially with capital letters. The second labeling scheme (**d**–**f**) is related to the number of bonds between the substituent and the –OH carbon. For example, the γ-position refers to 4-substituted phenol, 4,10-substituted pyrenol, and 2,7-substituted anthranol

The molecules considered in this study include symmetrically substituted halogenated phenols, 2-pyrenols, and 9-anthranols, as shown in Fig. 1. For ease of discussion, we have introduced a labeling scheme using capital letters to designate each possible substituent position that results in a symmetrically substituted compound. This labeling scheme is shown in Fig. 1a–c. For example, 2,6-difluorophenol would be designated A-fluorophenol. However, as will be discussed below, it was found to be advantageous to have an alternative labeling scheme that equates substituent positions with the number of bonds from the -OH substituted carbon. This labeling scheme is shown in Fig. 1d–f, and in this scheme, each position is indicated by a Greek letter. Both labeling schemes will be used in the remainder of the work. Considering symmetrical substituents with hydrogen, fluorine, chlorine, or bromine in each of the available positions led to 22 phenols, 94 pyrenols, and 94 anthranols for a total of 210 compounds considered in this study. Iodine-containing compounds were not considered in this study, as they would have required relativistic corrections in the calculations.

For all calculations, the B3LYP/6–311 + G(d,p) level of theory [28–36] was used on Gaussian 16 software [37]. The “nosymm” keyword was included in all calculations. Relaxed potential energy scans were performed by rotating the C–C-O–H dihedral angle from 0 to 360° in steps of 10°. The resulting rotational energy barrier was converted to kilojoule per mole (kJ/mol). When the two energy barriers (from 0 to 180° or from 180 to 360°) were unequal, the lower was chosen, as quantum tunneling will occur through the lower barrier rather than the higher one. Nuclear magnetic resonance (NMR) and natural bond order (NBO) calculations were performed after a geometry optimization at the B3LYP/6–311 + G(d,p) level of theory, and a frequency calculation was also done to ensure that the optimized structure was a local minimum as indicated by zero imaginary frequencies. NMR calculations were done on the optimized structure using gauge including atomic orbitals (GIAO) [38–42], and the least shielded eigenvalue (σ_11_) at the -OH substituted carbon was reported. NBO calculations [43–49] were done on the optimized structure, and the Wiberg bond index value of the carbon–oxygen bond was found by using the “pop = nboread” keyword with “$nbo bndidx $end” as the readable section at the end of the input file.

Machine learning was done using the classification and regression training (caret) package [50] in R version 4.4.0 [51]. The data were trained on a linear model. The lasso model and ridge regression model were also tried (data in supporting information) but did not result in improvement of the model. Descriptors used to create the model were the electronegativity, atomic radius [52], and Hammett constant [53] for the substituent in positions A-E. Data partitioning was done in two ways: the data was divided randomly into training and test data sets, with 30% of the data used as the test data and the remaining 70% of the data being used as the training set. Second, we used the leave-one-out (LOO) data partitioning method, in which a different linear model was created to predict the barrier height for each molecule, using all other molecules in the data set as the training set. The LOO method is expected to do a better job of predicting the test data, and it is commonly used with small data sets [54].

The R code and.csv files used to create the models are provided in the supporting information.

## Results and discussion

Substituents in aromatic compounds typically influence the properties and reactivity of these molecules through both resonance and inductive effects. However, both of these effects may be influenced by deviation from planarity of aromatic compounds. Importantly, we found that many of the substituted aromatic compounds considered in this study were not flat in their geometry-optimized forms. The inertial defect is a parameter used by spectroscopists to quantify the deviation from planarity of a molecule. The inertial defect is defined as I_c_-I_b_-I_a_ where I_a_, I_b_, and I_c_ are the three moments of inertia of the molecule. The inertial defect is zero for a planar molecule and the farther the inertial defect is from zero, the less planar the molecule. Inertial defects calculated from the rotational constants of the optimized structures are listed in Table 1.

**Table 1:** Inertial defect (amu A^2^) for the 210 compounds considered in this study

Substitution	F	Br	Cl
Phenols			
(None)		0.00	
A	0.00	− 0.01	0.00
AB	0.00	− 0.10	0.00
ABC	0.00	− 0.10	0.04
AC	0.00	0.06	0.01
B	0.00	− 0.02	0.01
BC	0.00	− 0.03	0.01
C	0.00	0.01	0.00
Pyrenols			
(None)		0.02	
A	− 0.05	0.19	− 0.03
AB	− 0.09	− 356.43	− 69.57
ABC	− 0.08	− 661.59	− 180.67
ABCD	− 0.06	− 2140.38	− 652.71
ABCDE	− 0.10	− 1412.99	− 819.22
ABCE	− 0.12	− 997.65	− 237.61
ABD	− 0.03	− 554.60	− 117.68
ABDE	− 0.13	− 625.99	− 132.70
ABE	− 0.08	− 473.33	− 81.91
AC	0.04	− 0.55	0.12
ACD	− 0.10	− 459.24	− 83.48
ACDE	− 0.04	− 788.95	− 189.52
ACE	− 0.13	− 0.50	− 0.23
AD	0.00	− 0.18	− 0.12
ADE	0.01	− 0.58	− 0.04
AE	0.08	0.31	− 0.15
B	− 0.05	0.07	− 0.01
BC	− 0.04	− 0.14	0.05
BCD	− 0.08	− 667.34	− 171.68
BCDE	− 0.09	− 921.56	− 275.46
BCE	0.06	0.55	− 0.22
BD	− 0.02	− 0.49	− 0.10
BDE	0.00	0.63	0.25
BE	− 0.03	− 0.29	0.14
C	0.01	0.12	− 0.09
CD	0.04	− 311.92	− 46.87
CDE	− 0.01	− 527.02	− 139.18
CE	− 0.08	0.34	− 0.07
D	− 0.02	− 0.24	0.04
DE	0.07	− 0.04	0.03
E	− 0.02	0.00	− 0.10
Anthranols			
(None)		− 0.06	
A	0.00	− 2.26	− 0.04
AB	0.01	− 27.56	− 4.37
ABC	− 0.04	− 41.23	− 7.91
ABCD	− 0.08	− 82.10	− 21.97
ABCDE	− 0.04	− 632.14	− 204.26
ABCE	− 0.13	− 62.71	− 21.91
ABD	− 0.09	− 46.93	− 13.34
ABDE	0.08	− 450.43	− 168.90
ABE	0.08	− 45.05	− 16.82
AC	− 0.03	− 1.51	0.08
ACD	− 0.09	− 16.43	0.40
ACDE	0.07	− 565.80	− 184.94
ACE	− 0.10	− 22.26	− 0.60
AD	− 0.03	− 14.53	0.03
ADE	0.05	− 480.14	− 156.46
AE	− 0.01	− 18.81	− 2.84
B	− 0.13	− 0.12	− 0.01
BC	− 0.39	− 1.16	− 0.22
BCD	− 0.21	− 3.14	− 1.15
BCDE	− 0.95	− 461.35	− 128.35
BCE	− 0.37	− 2.45	− 0.18
BD	− 0.28	− 2.11	− 0.57
BDE	− 0.69	− 358.85	− 95.57
BE	− 0.28	− 0.34	− 0.45
C	− 0.11	0.12	− 0.12
CD	− 0.07	0.29	− 0.09
CDE	− 0.40	− 449.70	− 124.92
CE	− 0.13	− 0.42	− 0.22
D	− 0.08	− 0.40	− 0.26
DE	− 0.33	− 357.29	− 95.54
E	− 0.11	− 0.12	− 0.09

**Fig. 2:**
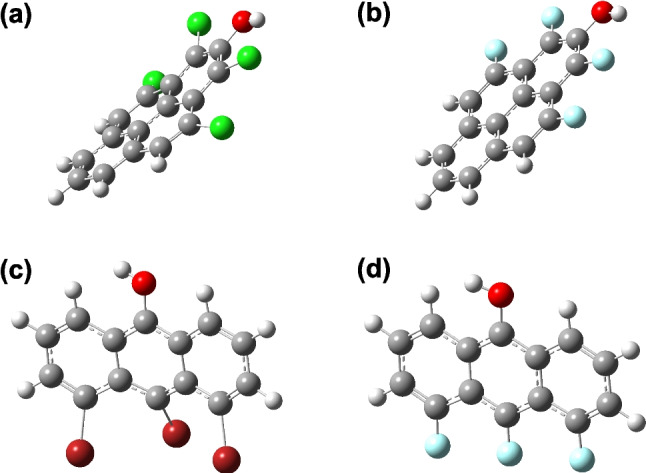
Some example geometry-optimized structures of pyrenols and anthranols that deviate from planarity or remain flat. **a** 1,3,4,10-Tetrachloropyren-2-ol, inertial defect =  − 69.6; **b** 1,3,4,10-tetrafluoropyren-2-ol, inertial defect =  − 0.09; **c** 4,5,10-tribromoanthran-9-ol, inertial defect =  − 357; **d** 4,5,10-trifluoroanthran-9-ol, inertial defect =  − 0.33

From Table 1, it can be seen that substituted phenols, as well as fluorine-substituted pyrenols and anthranols, remain essentially flat. However, addition of the larger bromine and chlorine substituents can lead to a large deviation from planarity of these structures. Pyrenols are nonplanar when there is a Br or Cl substituent at both the A and B positions and/or both the C and D positions. Similarly, anthranols deviate from planarity when there is a Br or Cl substituent at both the D and E positions. Structures of some planar and nonplanar geometry-optimized structures are shown in Fig. 2.

Before exploring whether machine learning could be used to predict barrier heights to rotation about the C-O bond in substituted aromatic alcohols, we explored correlations between calculated barrier heights and either the calculated NBO Wiberg bond index for the C-O bond or the σ_11_ component of NMR shielding at the -OH substituted carbon. The Wiberg bond index describes the single- or double-bond character of the C-O bond, and as such would be expected to correlate with the energy required to rotate about this bond. The more double-bond character the C-O bond has, the harder it would be to rotate about the bond, since it would require breaking the π-bond of the double bond [55]. Bond indices calculated for the substituted aromatic compounds considered in this work ranged from 1.0 to 1.10, indicating that the C-O bond retained mostly single-bond character.

The σ_11_ component of NMR shielding at the -OH substituted carbon was also calculated and compared to the barrier height for rotation about the C-O bond since the σ_11_ component of NMR shielding was previously found to correlate with the amount of π-electron density at the carbon for which it is calculated [56]. This parameter was explored in case the barrier height could be related to the amount of π-electron density present at the -OH substituted carbon.

**Fig. 3:**
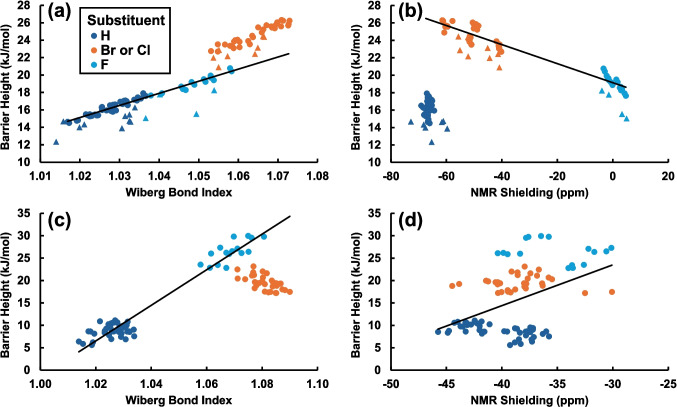
Correlations between calculated NBO Wiberg bond index, σ_11_ component of NMR shielding at the –OH carbon, and calculated rotational energy barrier heights. **a**, **b** Phenols (triangles) and pyrenols (circles) and **c**, **d** anthranols. The substituent in the A position is indicated by the color of the marker. Trendlines are shown as follows: **a** pyrenols with H or F at the A position, *R*^2^ = 0.972; **b** pyrenols with Br, Cl, or F at the A position, *R*^2^ = 0.933; **c** anthranols with H or F at the A position, *R*^2^ = 0.9445; and **d** all anthranols, *R*.^2^ = 0.1918

The results of these comparisons are shown in Fig. 3. Figure 3a indicates that for phenols and pyrenols, there is a good correlation between calculated bond index and barrier height to rotation. The barrier height increases with increasing bond index, as expected. Compounds with a Br or Cl substituent at the A position deviate slightly from this line. The slight increase in barrier height for these molecules may indicate a favorable interaction between the -OH group and either Br or Cl when the dihedral angle is zero. This would lead to a lowering of the energy at dihedral angles of 0° and 180°, thus increasing the barrier to rotation. Data for the phenols is similar to that of the pyrenols, but with the phenols having a slightly lower barrier height. The trendline shown on the figure is a linear correlation between pyrenols with either an H or F at the A position and indicates a good correlation with *R*^2^ of 0.972.

From Fig. 3b, for substituted phenols and pyrenols, a negative correlation is observed between NMR shielding and barrier height to rotation, but compounds with H at the A position do not follow this trend. Thus, the trendline shown includes pyrenols with F, Cl, or Br at the A position. This trend indicates that more π-electron density at the -OH carbon leads to a weaker C-O bond. As expected, shielding is strongly correlated with electronegativity of the substituent at the closest position to the -OH carbon.

Correlations between Wiberg bond index and barrier height to rotation for anthranols are shown in Fig. 3c. Again, there is a correlation between bond index and barrier height for the H- or F-substituted compounds, with compounds with a Br or Cl substituent in the A position deviating from this line. The trendline for anthranols with H or F at the A position again indicates a good correlation, with *R*^2^ of 0.9445. As opposed to the case for phenols and pyrenols, in the case of anthranols, compounds with a Br or Cl at the A position have a lower barrier height than would be predicted by the trendline. This difference may reflect the fact that the A position for the anthranols is two bonds away from the -OH substituted carbon, instead of one bond away in phenols and pyrenols.

Last, Fig. 3d shows correlations between NMR shielding and barrier height to rotation for anthranols. Here, we see that there is little correlation between the two parameters. This may reflect the fact that many of the substituted anthranols deviate from planarity in their optimized configurations. Although the Wiberg bond index and potentially the π-electron density at the substituted carbon would be expected to be correlated with the barrier height to rotation about the C-O bond, it is clear from Fig. 3 that these parameters and correlations are not sufficient to predict the rotational energy barrier. Therefore, we explored whether machine learning could be used to train a linear model to be able to predict the results of the quantum mechanical calculations of the barrier heights.

**Fig. 4:**
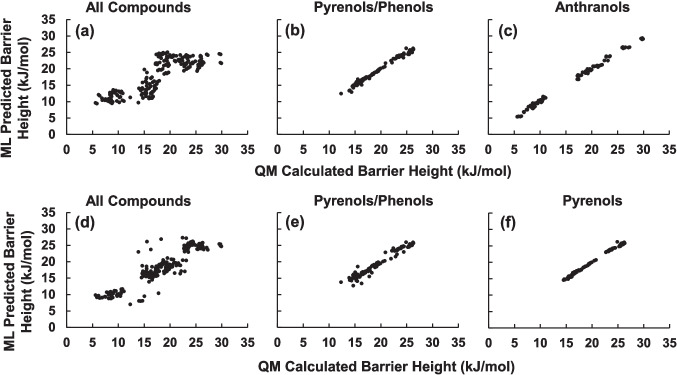
Comparison between quantum mechanical (QM) calculated and machine learning (ML) predicted barrier height to rotation about the C-O bond in substituted aromatic alcohols using LOO data partitioning. **a**, **b** Capital letter numbering scheme, **d**, **e** Greek letter numbering scheme, **c**, **f** identical results for both numbering schemes, **a**, **d** all compounds, **b**, **e** pyrenols and phenols, **c** anthranols, and **f** pyrenols

Figure 4 compares the results of the machine learning (ML) model to the quantum mechanical (QM) results. For the models shown in Fig. 4, the leave-one-out (LOO) data partitioning method was used. Similar results obtained by dividing the data set randomly into test (30%) and training (70%) sets are shown in Figure S1 of the supporting information. Correlation coefficients (*R*^2^) and RMS errors (RMSE) for all models are listed in Table 2.

**Table 2:** Correlation coefficients and root mean square errors (RMSE) for comparison between QM calculated and ML predicted rotational energy barriers

Capital letters	*R*^2^	RMSE (kJ/mol)	Capital letters	*R*^2^	RMSE (kJ/mol)
LOO all	0.6844	3.32	70–30 all	0.6840	3.28
LOO pyrenols/phenols	0.9889	0.418	70–30 pyrenols/phenols	0.9919	0.409
LOO pyrenols	0.9947	0.285	70–30 pyrenols	0.9949	0.288
LOO anthranols	0.9953	0.485	70–30 anthranols	0.9959	0.479
Greek letters	*R*^2^	RMSE (kJ/mol)	Greek letters	*R*^2^	RMSE (kJ/mol)
LOO all	0.8316	2.42	70–30 all	0.8090	2.80
LOO pyrenols/phenols	0.9595	0.799	70–30 pyrenols/phenols	0.9700	0.724
LOO pyrenols	0.9947	0.285	70–30 pyrenols	0.9945	0.298
LOO anthranols	0.9953	0.485	70–30 anthranols	0.9941	0.546

When the machine learning model was trained on all compounds considered in this study, using as descriptors the electronegativity, atomic radius, and Hammett constant of the substituents at positions A-E, comparing the predicted versus calculated barrier heights resulted in the plot shown in Fig. 4a. A better prediction could be achieved if we instead separated the compounds into phenols/pyrenols or anthranols and trained the machine learning model on the two groups separately. This resulted in the plots shown in Fig. 4b and c. When dividing the compounds into two groups, the machine learning model can predict rotational energy barrier heights with a root mean square error (RMSE) less than 0. 5 kJ/mol and *R*^2^ > 0.98. It is not unexpected that training the machine learning model on the pyrenols and phenols separately from the anthranols results in a better model, since the substituent closest to the -OH group in position A is expected to have the greatest influence on the rotational energy barrier, and the A-position is ortho to the -OH carbon in pyrenols and phenols but meta to the -OH carbon in anthranols.

This indicates that the capital letter labeling scheme shown in Fig. 1a–c may not be fully capturing the correct equivalence between substituent positions in the phenols, pyrenols, and anthranols considered in this study. Therefore, we have adopted a different labeling scheme in which ortho, meta, and para substituents are considered similarly in the model. In the Greek letter labeling scheme shown in Fig. 1d–f, substituent positions are designated by how many bonds they are from the -OH substituted carbon. For example, all substituents at the γ-position are exactly three bonds away from the -OH substituted carbon. It is expected that machine learning models trained using this labeling scheme will be able to better predict the barrier height to rotation for all of the compounds considered in this study.

Results of training the machine learning model on all compounds in this study using the Greek letter labeling scheme are shown in Fig. 4d. The agreement between QM calculated and ML predicted barrier heights improves slightly (*R*^2^ increases from 0.6844 to 0.8316 and RMSE decreases from 3.32 to 2.42 kJ/mol) but does not reach the level of accuracy obtained by separating the pyrenols and phenols from the anthranols shown in Fig. 4b and c. Interestingly, when a machine learning model is trained on the pyrenols/phenols group using the Greek letter labeling scheme, the agreement is slightly worse than when the capital letter labeling scheme is used. From the plot in Fig. 4e, it is clear that the Greek letter labeling scheme does not fully capture the equivalence of substituent positions between pyrenols and phenols. When the ML model is trained on only the pyrenols, as shown in Fig. 4f, the agreement is again excellent. The Greek labeling scheme is not perfect, either, since the ε and η positions in anthranol can also be considered to be γ-substituents, since both of these positions are three bonds away from the -OH substituted carbon. Thus, neither of our two labeling schemes fully capture the equivalence and difference between the different substituent positions in anthranols, pyrenols, and phenols. It is important to note that when training the machine learning model on only the anthranols (Fig. 4c) or only on the pyrenols (Fig. 4f), the two labeling schemes are equivalent, and the results are identical.

The coefficients in front of the different parameters in the linear model can also give insight into which parameters are important in determining the barrier height to rotation and how they contribute. The average coefficients and their standard deviations are listed in Tables 3 and 4 for the capital letter and Greek letter labeling schemes, respectively. In both cases, the LOO data partitioning was used and the averages and standard deviations are taken over the different models that are generated as each data point is sequentially left out.

**Table 3:** Coefficients in linear model resulting from machine learning using LOO data partitioning and capital letter labeling scheme

Coefficients (standard deviation), kJ/mol				
Parameter	All	Pyrenols/phenols	Anthranols	Pyrenols
E(A)	3.03 (0.04)	− 0.02 (0.01)	7.4 (0.1)	0.094 (0.009)
E(B)	− 0.66 (0.04)	0.372 (0.008)	− 1.38 (0.02)	0.565 (0.009)
E(C)	− 1.78 (0.06)	− 0.69 (0.01)	− 1.1 (0.2)	0.8 (0.1)
E(D)	− 0.02 (0.04)	0.475 (0.009)	0.01 (0.02)	0.55 (0.09)
E(E)	− 0.86 (0.05)	− 0.054 (0.009)	− 1.71 (0.02)	− 0.180 (0.008)
AR(A)	0.018 (0.001)	0.046 (0.001)	− 0.049 (0.005)	0.0386 (0.0009)
AR(B)	0.010 (0.002)	0.0222 (0.0006)	− 0.0018 (0.001)	− 0.0057 (0.0009)
AR(C)	− 0.057 (0.002)	− 0.0343 (0.0006)	− 0.032 (0.006)	0.008 (0.004)
AR(D)	− 0.108 (0.001)	0.0021 (0.0003)	0.010 (0.001)	0.006 (0.003)
AR(E)	0.075 (0.003)	− 0.0104 (0.0008)	0.018 (0.001)	0.0025 (0.0008)
H(A)	18.6 (0.3)	22.9 (0.3)	19 (1)	25.2 (0.2)
H(B)	0.5 (0.4)	− 0.9 (0.1)	2.7 (0.4)	7.4 (0.3)
H(C)	15.6 (0.4)	8.7 (0.1)	8 (1)	− 2.6 (0.9)
H(D)	24.6 (0.2)	− 0.00 (0.08)	− 8.0 (0.3)	− 0.9 (0.8)
H(E)	− 22.0 (0.9)	3.0 (0.2)	− 4.1 (0.3)	− 0.4 (0.2)
Constant	15.5 (0.2)	13.75 (0.07)	6.8 (0.7)	9.0 (0.4)

*E* electronegativity, *AR* atomic radius/picometers, *H* Hammett constant

The resulting linear model for all compounds is 3.03*E(A) − 0.66*E(B) − 1.78*E(C) + … − 22.0 *H(E) + 15.5

**Table 4:** Coefficients in linear model resulting from machine learning using LOO data partitioning and Greek letter labeling scheme

Coefficients (standard deviation), kJ/mol				
Parameter	All	Pyrenols/phenols	Anthranols	Pyrenols
E(α)	− 0.21 (0.06)	− 0.02 (0.02)		0.094 (0.009)
E(β)	3.6 (0.3)	0.6 (0.3)	7.4 (0.1)	
E(γ)	− 0.59 (0.04)	0.16 (0.02)	− 1.38 (0.02)	0.565 (0.009)
E(δ)	1.46 (0.05)	1.0 (0.2)	− 1.1 (0.2)	0.8 (0.1)
E(ε)	0.08 (0.04)		− 0.01 (0.02)	
E(ζ)	3.1 (0.3)	0.8 (0.2)		0.55 (0.09)
E(η)	− 0.64 (0.03)	− 0.08 (0.01)	− 1.71 (0.02)	− 0.180 (0.008)
AR(α)	0.045 (0.003)	0.047 (0.001)		0.0386 (0.0009)
AR(β)	− 0.094 (0.009)	0.023 (0.008)	− 0.049 (0.005)	
AR(γ)	− 0.009 (0.002)	− 0.003 (0.001)	− 0.018 (0.001)	− 0.0057 (0.0009)
AR(δ)	0.030 (0.002)	0.013 (0.005)	− 0.032 (0.006)	0.008 (0.004)
AR(ε)	0.014 (0.002)		0.010 (0.001)	
AR(ζ)	0.072 (0.009)	0.011 (0.005)		0.006 (0.003)
AR(η)	− 0.001 (0.002)	− 0.005 (0.001)	0.018 (0.001)	0.0025 (0.0008)
H(α)	25 (1)	22.8 (0.5)		25.2 (0.2)
H(β)	32 (2)	− 2 (2)	19 (1)	
H(γ)	5.2 (0.7)	6.0 (0.4)	2.7 (0.4)	7.4 (0.3)
H(δ)	− 8.8 (0.5)	− 4 (1)	8 (1)	− 2.6 (0.9)
H(ε)	− 10.0 (0.7)		− 8.0 (0.3)	
H(ζ)	− 19 (2)	− 2 (1)		− 0.9 (0.8)
H(η)	− 0.6 (0.5)	1.5 (0.4)	− 4.1 (0.3)	− 0.4 (0.2)
Constant	4.5 (0.9)	8.9 (0.8)	6.8 (0.7)	9.0 (0.4)

*E* electronegativity, *AR* atomic radius/picometers, *H* Hammett constant

As can be seen in Tables 3 and 4, the coefficients differ greatly among the different models and between the different labeling schemes. One might expect that the coefficients in a linear model would be largest for the position closest to the -OH substituted carbon, indicating that the substituent in this position is the most important when determining the rotational energy barrier about this C-O bond. However, from the coefficients in Tables 3 and 4, this is not always the case. The Hammett constant in position A or position α is generally large, but in the capital letter labeling scheme, Hammett constants of the substituent in positions C, D, and E are also important in the linear model for all compounds. Electronegativity of the substituent in the β and ζ positions is important in the models for all compounds, pyrenols/phenols, and anthranols, but less important in the model for pyrenols alone. The coefficient for atomic radius is largest for the A position in the models for pyrenols/phenols, anthranols, and pyrenols, but not in the model for all compounds. The fact that the coefficients are not always greater for substituent positions closest to the -OH carbon may be related to the deviation from planarity of different substituted aromatic compounds. Since positions C, D, and E influence the planarity of the compound, large coefficients in front of parameters for these positions, especially the atomic radius, could indicate that deviation from planarity is correlated with energy required to rotate about the C-O bond. Deviation from planarity would be expected to decrease the aromaticity of these compounds, which would influence how different substituents affect the rotational energy barrier.

Although the compounds considered this far only contained one type of substituent (either F, Cl, or Br), we wanted to test whether the ML model could accurately predict rotational energy barriers in symmetrical aromatic alcohols with mixed substituents. For the test data set, we randomly created several anthranols, phenols, and pyrenols with mixed substituents. The rotational energy barrier for each molecule was predicted using the different machine learning models and compared to DFT-calculated rotational energy barrier heights. The machine learning model in each case used all the compounds with only one type of substituent as the training set. The results are shown in Table 5. The last row of the table contains the RMSE for the compounds with mixed substituents. The performance of each ML model in compounds with mixed substituents is similar to that of the test set containing compounds containing only one kind of substituent. This indicates that these models have broader applicability than just molecules in the test set. Non-symmetric compounds with mixed substituents could be considered in future work.

**Table 5:** Rotational energy barrier heights for compounds with mixed substituents

Compound	Substituent at position “X”					Barrier height	Capital letters	Greek letters	Capital letters	Greek letters	Anthranols	Pyrenols
	A	B	C	D	E	QM	All compounds	All compounds	Pyrenols/phenols	Pyrenols/phenols		
Anthranol	Br	Cl	F	F	H	19.534	21.435	19.847			18.514	
Anthranol	Br	H	F	H	H	21.004	23.628	19.745			20.510	
Anthranol	Br	H	F	F	H	21.582	21.376	19.775			20.342	
Anthranol	H	Cl	H	F	H	9.426	10.304	11.048			9.306	
Anthranol	H	Cl	H	F	Br	8.507	10.376	10.319			8.439	
Phenol	Br	H	F			20.138	19.483	16.671	21.208	22.378		
Phenol	H	Cl	Br			14.939	13.691	18.308	14.510	15.632		
Phenol	F	Br	H			17.906	23.207	19.435	18.806	17.449		
Phenol	F	H	Cl			16.698	21.161	11.312	16.407	17.288		
Pyrenol	H	Br	Br	H	Cl	16.672	11.965	15.496	16.921	16.545		16.605
Pyrenol	Cl	H	Br	H	H	23.052	21.154	24.701	23.245	23.439		23.295
Pyrenol	F	H	Br	Cl	H	18.956	22.345	19.174	18.632	18.781		18.595
Pyrenol	H	F	Br	Cl	F	17.223	14.102	14.248	16.821	16.170		16.603
Pyrenol	F	F	Cl	Cl	H	20.348	21.695	18.162	20.196	19.423		19.933
RMSE (kJ/mol)							2.85	2.44	0.539	0.955	0.754	0.387

## Conclusions

Quantum mechanical calculations were used to determine the barrier height for rotation about the C-O bond for a series of halogenated phenols, pyrenols, and anthranols. Machine learning was then used to train a linear model to predict these rotational energy barriers, based on the electronegativity, atomic radius, and Hammett constant of the substituent at each position. When the substituents were labeled sequentially in order from the -OH substituted carbon, it was found that the machine learning model did a better job of predicting the barrier heights when the model was trained on and applied separately to pyrenols/phenols and anthranols than when one model was trained on and applied to all compounds. When the substituent positions were grouped according to the number of bonds between the position and the -OH substituted carbon instead of labeled sequentially, the machine learning model that was trained on all compounds improved in performance, while the model that was trained on pyrenols and phenols actually performed slightly worse. Even though the models were trained on compounds containing only one kind of substituent, they performed similarly well on compounds containing mixed substituents, demonstrating the broad applicability of the models.

Coefficients in the linear model that were predicted by machine learning differed among the different models and between the two labeling schemes and assigned importance to parameters of substituents at positions that are not immediately obvious based on chemical intuition. The use of machine learning is important, since it was found that correlations between expected parameters such as Wiberg bond order or NMR shielding and the barrier height to rotation are not strong enough to develop predictive linear models. The results of this study indicate that machine learning can be used to predict chemical properties such as barrier height to rotation from simple molecular parameters. This kind of machine learning can be used in future studies to predict properties such as rotational energy barriers in other substituted aromatic compounds. The results also shed light on the inequivalences in different substituent positions between phenols, pyrenols, and anthranols. These results may also be used in future studies to understand and model extended conjugated carbon systems, such as graphene flakes with substituents at the edges.

## Supplementary Information

Machine learning results with 70–30 data partitioning and different prediction methods, R code and.csv files for performing the machine learning, Excel file containing the NMR and NBO results.

Below is the link to the electronic supplementary material.

Supplementary file1 (XLSX 18 KB)

Supplementary file2 (DOCX 86 KB)

Supplementary file3 (CSV 17 KB)

## Data Availability

The datasets generated and analyzed during the current study are available from the corresponding author on reasonable request. Geometry optimization output files have been deposited to a Mendeley Data set: Cerabona, Steven; Brown, Gordon; Casabianca, Leah (2025), “Supplemental Data for "Quantum Mechanical and Machine Learning Prediction of Rotational Energy Barriers in Halogenated Aromatic Alcohols"”, Mendeley Data, V1, 10.17632/z2hz9prrb2.1.
