# Supplementary material for: Quantum mechanical and machine learning prediction of rotational energy barriers in halogenated aromatic alcohols
Source: J Mol Model. 2025 Feb 24;31(3):93. doi: 10.1007/s00894-025-06321-y (PMC11850414; doi:10.1007/s00894-025-06321-y)
Supplement: Supplementary file 2 — Supplementary file2 (DOCX 86 KB) [file 894_2025_6321_MOESM2_ESM.docx]

Supporting information for:

**Quantum Mechanical and Machine Learning Prediction of Rotational Energy Barriers in Halogenated Aromatic Alcohols**

Steven T. Cerabona^(1)^, Gordon G. Brown^(2)^, Leah B. Casabianca*^(1)^

*^(1)^ Department of Chemistry, Clemson University, Clemson, SC, 29634 USA*

*^(2)^ South Carolina Governor’s School for Science and Mathematics, Hartsville, SC, 29550 USA*

**Machine Learning Results with 70-30 Data Partitioning**

Figure S1 shows a comparison between ML predicted and QM calculated rotational energy barriers. This figure is similar to Figure 4 in the main text, but instead of using LOO partitioning, the data partitioning was done by randomly dividing the data set into a test (30%) and training (70%) data set. Note that since the data partitioning is random, each run will generate a different model, but the plots below and fit parameters in Table 2 of the main text are representative. From the results in Table 2, the LOO and 70-30 data partitioning schemes perform similarly when generating the ML model.

***Figure S1.*** Comparison between quantum mechanical (QM) calculated and machine learning (ML) predicted barrier height to rotation about the C-O bond in substituted aromatic alcohols using 70-30 data partitioning. (a-b) capital letters numbering scheme, (d-e) Greek letters numbering scheme, (c, f) identical results for both numbering schemes, (a, d) all compounds, (b, e) pyrenols and phenols, (c) anthranols, (f) pyrenols.

**Machine Learning Results with Different Prediction Methods**

Different machine learning prediction methods were tested, including the lasso and ridge regression models. For the lasso and ridge regression models, the default tuning parameters were used, i.e. three values of lambda were tested for the ridge regression model. As can be seen from Figure S2, neither the lasso nor the ridge regression model significantly improved the results over the linear model. Advantages of the linear model over the other two models, however, include computational speed and ease of interpretation. Thus, the linear model was used in the rest of this paper.

***Figure S2.*** Comparison between quantum mechanical (QM) calculated and machine learning (ML) predicted barrier height to rotation about the C-O bond in substituted aromatic alcohols using different ML prediction methods. (a-c) capital letters numbering scheme, (d-f) Greek letters numbering scheme, (a, d) linear model, (b, e) lasso model, (c, f) ridge regression model. In each case, the LOO data partitioning method was used and all compounds were considered.

Table S1: Correlation Coefficients and Root Mean Square Errors (RMSE) for Comparison between QM Calculated and ML Predicted Rotational Energy Barriers for Different ML Prediction Methods

**R-Code for Generating Machine Learning Model – LOO:**

install.packages("caret")

install.packages("MuMIn")

library(caret)

library(MuMIN)

rm(list = ls())

aromatics_data <- read.csv("params_all_letters_final.csv")

nummolec=210;

#nummolec=dim(aromatics_data)[1]

numcolumns=dim(aromatics_data)[2]

BH=aromatics_data[1:nummolec,numcolumns]

numparams=numcolumns-1;

predictions <- rep (0, nummolec)

modelcoeffs=matrix(data=0,nrow=nummolec,ncol=numparams)

for (i in 1:nummolec) {

training <- aromatics_data[-i,]

testing <- aromatics_data[i,]

train_inputs=training[,2:numparams]

train_outputs=training[,numcolumns]

test_inputs=testing[,2:numparams]

model <- train(train_inputs, train_outputs, method="lm")

modelcoeffs[i,]<-coef(summary(model))[,1] #comment this line for ridge or lasso method

predictions[i] <- predict(model,test_inputs)

}

qplot (BH, predictions)

cor (BH,predictions)

results<-cbind(BH,predictions)

write.csv(results,"predictions_all_letters.csv")

write.csv(modelcoeffs,"model_coefficients_all_letters.csv")

**R-Code for Generating Machine Learning Model – 70-30:**

install.packages("caret")

install.packages("MuMIn")

library(caret)

library(MuMIN)

rm(list = ls())

aromatics_data <- read.csv("params_all_letters_final.csv")

nummolec=210;

#nummolec=dim(aromatics_data)[1]

numcolumns=dim(aromatics_data)[2]

BH=aromatics_data[1:nummolec,numcolumns]

numparams=numcolumns-1;

predictions <- rep (0, nummolec)

modelcoeffs=matrix(data=0,nrow=nummolec,ncol=numparams)

ind1 <- createDataPartition(y=BH,p=0.7,list=FALSE,times=1)

training <- aromatics_data[ind1,]

testing <- aromatics_data[-ind1,]

train_inputs=training[,2:numparams]

train_outputs=training[,numcolumns]

test_inputs=testing[,2:numparams]

test_outputs=testing[,numcolumns]

model <- train(train_inputs, train_outputs, method="lm")

predictions <- predict(model,test_inputs)

qplot (test_outputs, predictions)

cor (test_outputs, predictions)

results<-cbind(test_outputs, predictions)

write.csv(coef(summary(model)),"model_coefficients_all__letters_3070.csv")

write.csv(results,"predictions_all_letters_3070.csv")

**References**

<https://clemsonciti.github.io/rcde_workshops/r_machine_learning/00-index.html>.
